# Book Reviews

**Published:** 1894-07

**Authors:** 


### *BOOK REVIEWS.*

---

An American Text-Book of the Diseases of Children. By American Teachers, edited by Louis Starr, M.D., Physician to the Children's Hospital, Philadelphia, etc., assisted by Thos. S. Westcott, M.D., Philadelphia. W. B. Saunders, Publisher, 925 Walnut Street, Philadelphia.

This is an exceedingly good work, probably the best and most complete single-volume book on the diseases of children. Some of the most distinguished American teachers and authorities on pediatrics are among the contributors. Thus we may mention the introductory chapter by the editor, Dr. Starr, on the "Investigation of Disease and the General Management of Children." This chapter describes the physical examination, proper feeding, clothing and bathing of children, with some general remarks on the treatment of disease. Dr. A. R. Leeds, of New Jersey, describes the "Chemistry of Milk and Artificial Foods." Dr. E. P. Davis of Philadelphia, has a good chapter on "Injuries Incident to Birth and Diseases of the New-born." Dr. Osler, of Baltimore, furnishes an excellent section on "Tuberculosis," which is able and thorough. One of the best chapters is that on "Rachitis," by Dr. J. Lewis Smith, of New York. Some of the other chapters are "Diphtheria," by Dr. Dillon Brown, New York; "Tracheotomy and Intubation of the Larynx," by Dr. H. R. Wharton, Philadelphia; "Anemia," by Dr. F. A. Packard, Philadelphia; "Diseases of the Mouth," by Dr. Forchheimer, Cincinnati; "Diarrheal Diseases," by Dr. V. C. Vaughan, Ann Arbor; "Pneumonia," by Dr. William Pepper, Philadelphia; and many others by the most prominent writers. The Diseases of the Skin are described by Dr. W. A. Hardaway, St. Louis; Diseases of the Ear, by Dr. B. A. Randall, Philadelphia, and Diseases of the Eye, by Dr. G. E. DeSchweinitz, Philadelphia.

The above outline represents very imperfectly the scope of this excellent work. This is a work of many authors, and affords a good illustration of the superiority of the composite text-book over the book of one author. We take pleasure in recommending it as an encyclopedic work of great merit on the diseases of children.

---

TREATMENT OF DISEASES OF THE STOMACH AND INTESTINES. By Dr. Albert Mathieu, Physician to the Paris Hospitals. New York: William Wood & Co.

Facts brought forward in late years concerning especially gastro-intestinal pathology, and the studies made in the chemistry of the stomach and the effects of toxines in the alimentary tract have had a large influence upon the treatment of gastro-intestinal diseases. The object of this book is to define this new treatment in the light of our latest knowledge.

Thirty-seven pages of the work are devoted to the general principles of diagnostic technique, in which the methods of examining the stomach contents are carefully described. Part III. discusses the principal clinical forms of dyspepsia and the most common symptoms of gastro-intestinal diseases. Under this head, vomiting, disorders of the appetite, constipation, diarrhea, gastro-intestinal antisepsis, acute indigestion, etc., receive appropriate consideration. Part IV. relates especially to the treatment of gastro-intestinal diseases. In his treatment of them every allowance is made for the latest therapeutics, but the author is conservative and does not give undue prominence to measures still tentative. In appendicitis he advises a resort to opium and absolute rest before the knife is used. At the same time he points out with distinctness the character of cases in which the knife is indicated. This book is an excellent one which will both please and instruct the reader. An appendix of useful formulæ closes the volume.

---

ILLUSTRATED ENCYCLOPEDIC MEDICAL DICTIONARY.—Being a Dictionary of the Technical Terms used by Writers on Medicine and the Collateral Sciences, in the Latin, English, French and German languages. Edited by Frank P. Foster, M.D., New York. Volume IV. D. Appleton & Co., New York.

This is the final volume of this immense work beginning at *Minn* and going through with the remainder of the alphabet. The frontispiece is a handsome colored plate showing the normal and abnormal constituents of urine. It is impossible to make a minute and detailed review of a book of such size and nature, but we may say of the complete work that it is the greatest achievement in medical-dictionary-making that has ever been attempted. The editors and publishers deserve great credit and the thanks of the profession for placing at their command such a classic. The words defined cover a wide range of the sciences allied to medicine, such as botany, pharmacy and electro-therapeutics. The definitions are accurate and condensed, and wherever descriptions are attempted only such important points are mentioned as will make a clear impression upon the reader. In spite of such attempts at condensation we have here a work of more than three thousand pages, representing almost an endless amount of labor and energy on the part of the dozen or more editors. Other dictionaries will come and go, but for many years, at least, this one will be unexcelled and accepted as our highest authority.

---

OPERATIVE SURGERY. By Th. Kocher, M.D., Professor at the University and Director of the Surgical Clinic at the Berne University. William Wood & Co., New York.

This is a very compact and practical work on operative surgery, by one of the most distinguished of European surgeons. The first part goes very fully into the methods of aseptic and antiseptic surgery, antiseptic agents, disinfection, the choice of anesthetics, etc. Part II. describes the various operations on the skin, the neck, spinal column, thorax, abdomen, perineum, etc., with ligation of the arteries, removal of the appendix, suture of the intestines, herniotomy, cholecystotomy, operations on nerves, etc. The next part describes excisions and resections of different bones and joints. Part IV. is devoted to amputations and exarticulations.

The author gives in condensed shape what he considers to be the best methods of operating, without describing with detail and confusion the entire list of methods in use. His book is the latest, and one of the best, on the subject.

DISEASES OF THE SKIN. AN OUTLINE OF THE PRINCIPLES AND PRACTICE OF DERMATOLOGY. By Malcolm Morris, Surgeon to the Skin Department, St. Mary's Hospital, London, etc., etc. With eight chromo-lithographs and seventeen wood-cuts. Philadelphia: Lea Bros. & Co. 1894.

This is a small work (necessarily condensed) by an able dermatologist. The illustrations are as good as the average. As with the majority of dermatological pictures, they require a label for identification. A few of them really show the disease. The author intends that the book shall present clinical facts, and the modern accepted etiology. His classification is based upon "proved or probable, etiological affinities." The work is well up to date, and will doubtless rank among the best of its kind. M. B. H.

---

A PRACTICAL TREATISE ON THE DISEASES OF THE HAIR AND SCALP. By George Thomas Jackson, M.D., Professor of Dermatology Woman's Medical College, New York Infirmary, etc., etc. New, revised and enlarged edition. New York. E. B. Treat. Price, $2.75.

This edition is an improvement upon the first. The literature has been brought up to January, 1893, and nine new illustrations have been added. The work is unique in its field, and is a book which should be useful to any one who desires a sensible and practical guide in the management of the hair and scalp. The anatomy, physiology and hygiene of the hair are first given, then the various anomalies and diseases of the hair and scalp. Much is to be found in this work which is not published in books on dermatology. Dr. Jackson is a careful and thorough author, and his name alone should do much to recommend a work. M. B. H.

---

WE have received the Transactions of the American Orthopedic Association for the Seventh Session, held in St. Louis, September, 1893. It includes a list of very interesting papers, relating mainly to diseases of the spine, hip, knee, and the varieties of club-foot. The President of the Association is Dr. A. M. Phelps, of New York; Secretary, Dr. John Ridlon, 103 State street, Chicago.

